# Systemic immuno-metabolic inflammatory indices (neutrophil-to-HDL cholesterol ratio and systemic inflammation response index) are strongly associated with metabolic dysfunction-associated steatotic liver disease: a propensity score-matched study

**DOI:** 10.3389/fimmu.2026.1768302

**Published:** 2026-03-09

**Authors:** Huimin Liang, Boqiao Wang, Deyuan Zhang, Zhongzhong Zhang, Xu Chen, Yijia Xu, Chengwei Zhang, Jinyang Li, Jie Yang, Pengjiao Xi, Jianwei Zhang, Yan Zhang, Haize Ge

**Affiliations:** 1 School of Nursing, Tianjin Medical University, Tianjin, China; 2 Office of Science, Technology and Innovation Management, Tianjin Medical College, Tianjin, China; 3 Department of Hepatobiliary and Pancreatic Surgery and Ophthalmic Nursing Unit, The Eighth Affiliated Hospital of Sun Yat-sen University, Shenzhen, China; 4 School of Life Sciences, East China Normal University, Shanghai, China; 5 Department of Clinical Laboratory, Tianjin University Central Hospital, The Third Central Hospital of Tianjin, Tianjin Key Laboratory of Extracorporeal Life Support for Critical Diseases, Artificial Cell Engineering Technology Research Center, Tianjin Institute of Hepatobiliary Disease, Tianjin, China; 6 Department of Clinical Laboratory Diagnostics, Tianjin Medical University, Tianjin, China; 7 Department of Infection, Shanxi Bethune Hospital, Shanxi Academy of Medical Sciences, Tongji Shanxi Hospital, Third Hospital of Shanxi Medical University, Taiyuan, China

**Keywords:** immuno-metabolic inflammation, metabolic dysfunction-associated steatotic liver disease, neutrophil-to-HDL cholesterol ratio, systemic immune dysregulation, systemic inflammation response index

## Abstract

**Background:**

Metabolic Dysfunction-Associated Steatotic Liver Disease (MASLD) is increasingly recognized as a systemic immuno-metabolic disorder characterized by chronic low-grade inflammation and dysregulated innate immune activation. Circulating immune-related indices derived from routine blood tests may reflect early immuno-metabolic imbalance and provide accessible markers for identifying disease-associated immune dysregulation.

**Aim:**

To investigate the associations of two systemic immuno-metabolic inflammatory indices, namely the neutrophil-to-high-density lipoprotein cholesterol ratio (NHR) and the systemic inflammation response index (SIRI) with MASLD, and to evaluate their combined discriminative performance.

**Methods:**

In this retrospective case–control study, adult participants attending a tertiary hospital between November 2023 and October 2024 were enrolled. After strict eligibility screening, propensity score matching (1:1) was applied to balance demographic characteristics and comorbidities, yielding 522 matched individuals (261 with MASLD and 261 without MASLD). Multivariable conditional logistic regression was used to assess associations between immuno-metabolic inflammatory indices and MASLD. Discriminatory performance was assessed using receiver operating characteristic (ROC) curves. The DeLong test, Net Reclassification Improvement (NRI), and Integrated Discrimination Improvement (IDI) were used to evaluate discriminative ability. Model calibration was evaluated using the Hosmer-Lemeshow test, Brier score, and calibration curves. Internal validation was performed using 2,000 bootstrap resamples.

**Results:**

After propensity score matching, both NHR and SIRI were independently associated with MASLD in multivariable analysis. The combined model integrating NHR, SIRI, and alanine aminotransferase (ALT) demonstrated optimal discriminative performance (AUC = 0.865, sensitivity = 72.41%, specificity = 84.30%), significantly outperforming the ALT-only model (DeLong test, Z = -8.3083, *P* < 0.001). The total Net Reclassification Improvement (NRI) was 0.345 (95% CI: 0.245–0.438), and the total Integrated Discrimination Improvement (IDI) was 0.348 (95% CI: 0.282–0.424, *P* < 0.001). The Hosmer-Lemeshow test (χ² = 11.043, df = 8, *P* = 0.199) and Brier score (0.151, 95% CI: 0.134–0.168) confirmed good model calibration, and the calibration curve showed excellent agreement between predicted probabilities and actual incidence. Internal validation through 2,000 bootstrap resamples resulted in a corrected AUC of 0.866 (95% CI: 0.836–0.897), with an average optimism of -0.001, indicating good model stability and minimal risk of overfitting.

**Conclusion:**

Systemic immuno-metabolic inflammatory indices reflecting innate immune activation and lipid-related anti-inflammatory dysfunction (NHR and SIRI) are closely associated with MASLD. The combined assessment of NHR, SIRI, and ALT provides a robust, clinically accessible approach with significant incremental value for identifying immuno-metabolic risk in MASLD, highlighting the central role of systemic immune dysregulation in metabolic liver disease.

## Graphical Abstract

**Figure:**
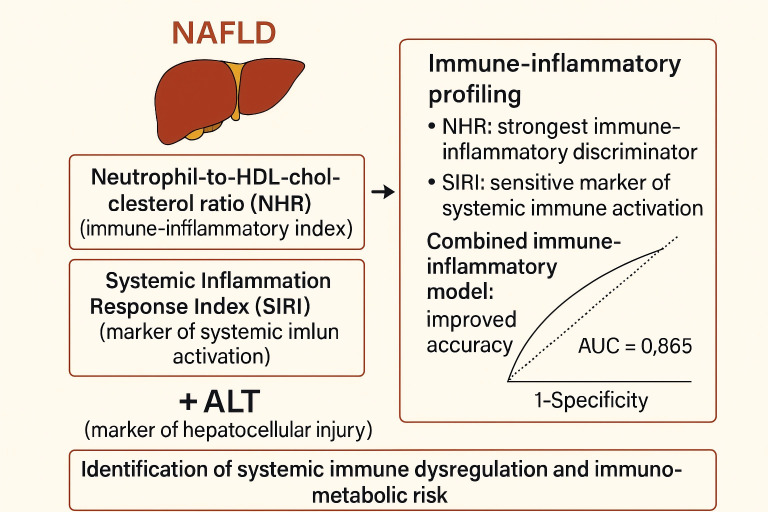
Diagnostic contribution of NHR, SIRI, and ALT to MASLD identification. This schematic summarizes the diagnostic roles of the neutrophil-to-HDL-cholesterol ratio (NHR), the Systemic Inflammation Response Index (SIRI), and alanine aminotransferase (ALT) in Metabolic Dysfunction-Associated Steatotic Liver Disease (MASLD). NHR demonstrated the strongest discriminative capacity among single biomarkers, whereas SIRI showed favorable sensitivity. Integrating these indices into a combined model further improved diagnostic accuracy, yielding an AUC of 0.865 and supporting their application in early screening and risk stratification.

## 1 Introduction

Metabolic Dysfunction-Associated Steatotic Liver Disease (MASLD) has become the most prevalent chronic liver condition worldwide and represents a major global health burden (1, 2). Traditionally regarded as a liver-restricted disorder, MASLD is now increasingly recognized as a systemic immuno-metabolic disease (3). Chronic low-grade inflammation and persistent immune activation play central roles in its initiation and progression (4, 5). Metabolic stress associated with obesity, insulin resistance, and dyslipidemia triggers immune dysregulation, which extends beyond the liver and leads to systemic inflammatory imbalance, further exacerbating metabolic abnormalities (6, 7).

Accumulating immunological evidence suggests that innate immune activation is a key driver of metabolic liver disease (8). Neutrophils and monocytes respond rapidly to metabolic stress and mediate inflammation signaling through the release of reactive oxygen species, pro-inflammatory cytokines, and neutrophil extracellular traps. This amplification of hepatic inflammation induces hepatocyte injury and activates hepatic stellate cells (9, 10). Meanwhile, adaptive immune regulation, characterized by changes in lymphocyte subpopulations (such as a reduction in regulatory T cells), is impaired, failing to effectively suppress uncontrolled innate immune activation. This results in persistent inflammation that drives disease progression from simple hepatic steatosis to non-alcoholic steatohepatitis (NASH) and eventually cirrhosis (11). The imbalance between excessive innate immune activation and impaired adaptive immune function forms a vicious cycle of “inflammation-injury-fibrosis,” which is central to the pathogenesis of MASLD.

Lipid metabolism also plays an crucial immunomodulatory role in MASLD. High-density lipoprotein (HDL) not only participates in cholesterol transport but also exerts anti-inflammatory and antioxidant effects (12). HDL can limit neutrophil activation, reduce immune cell adhesion, and block the activation of pro-inflammatory signaling pathways (13). In states of metabolic dysfunction, both the quantity and function of HDL are often impaired, weakening its protective immunoregulatory effects (14). The interaction between activated neutrophils and dysfunctional HDL forms a key immuno-metabolic axis that reflects systemic inflammatory stress and serves as a potential target for MASLD assessment. This core mechanism is not unique to MASLD, it also exists in other inflammation-related metabolic diseases such as metabolic syndrome and type 2 diabetes (T2DM), serving as a common pathological basis linking these three conditions (15–17).

The neutrophil-to-high-density lipoprotein cholesterol ratio (NHR) precisely captures the core imbalance of “inflammatory activation - anti-inflammatory deficiency”, reflecting the combined effects of innate immune activation and impaired anti-inflammatory capacity (18). It demonstrates clear pathophysiological significance across the three diseases, providing a solid mechanistic foundation for focusing research on MASLD. In metabolic syndrome, an elevated NHR directly reflects the synergistic effects of neutrophil-mediated systemic inflammation and HDL’s impaired anti-inflammatory function, strongly correlating with cardiovascular metabolic risk markers such as insulin resistance (HOMA-IR) and triglyceride/HDL-C ratio. In T2DM, NHR serves as an important predictor of disease onset and progression, and it is closely related to the incidence of MASLD in T2DM patients. Large-scale population studies show that NHR is independently positively correlated with both MASLD and significant liver fibrosis.

The Systemic Inflammation Response Index (SIRI), calculated from neutrophil, monocyte, and lymphocyte counts, reflects the balance between innate immune activation and adaptive immune regulation (19). This imbalance is also a common inflammatory feature shared by the three diseases. In MASLD, SIRI is closely related to the degree of hepatic steatosis and inflammatory activity, and it independently reflects disease severity beyond traditional metabolic indicators, further supporting its central role in the inflammation-metabolism axis (20–23).

Although the clinical value of NHR and SIRI has been preliminarily confirmed, existing studies have not clarified their synergistic and complementary effects, nor have they systematically explained how they collaboratively cover the core pathological pathway of MASLD, namely “neutrophil activation - HDL dysfunction - immune imbalance” (24–30). Particularly crucial is whether these immune-related indices, which integrate both inflammatory and metabolic characteristics, can provide supplementary value to traditional liver enzyme tests, a question that has yet to be definitively validated. Therefore, this study focuses on the core pathological mechanisms of MASLD, systematically exploring the associations and clinical value of NHR and SIRI in the disease, with a clear focus on their combined effectiveness with traditional liver enzyme alanine aminotransferase (ALT) . The goal is to provide a convenient and efficient diagnostic and risk stratification tool for MASLD based on routine testing for clinical use.

## 2 Materials and methods

### 2.1 Study design and participants

**Figure 1:**
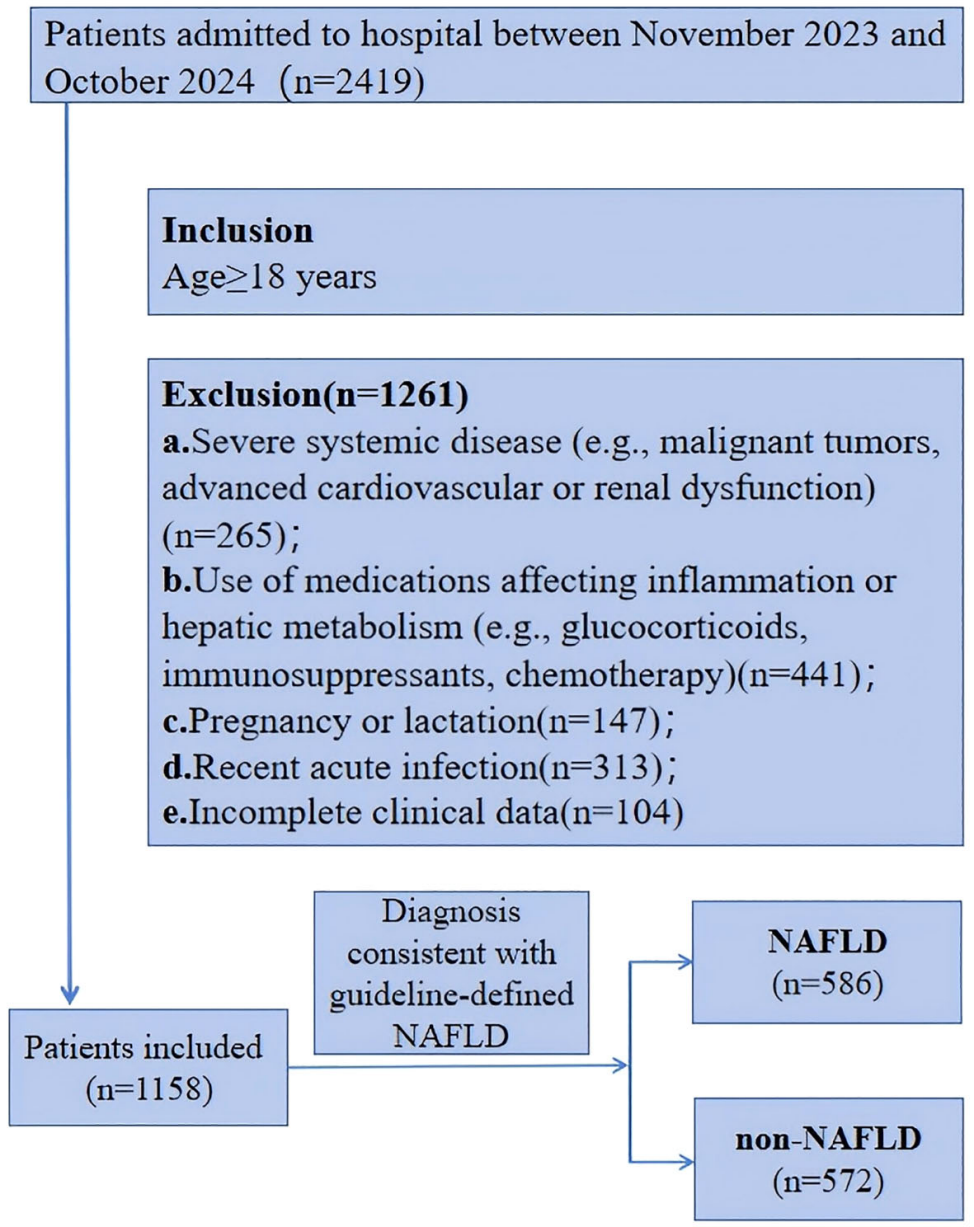
Flowchart of participant selection. The diagram illustrates the selection process for patients admitted between November 2023 and October 2024. Of the 2,419 individuals screened, 1,261 were excluded due to severe systemic disease, use of medications affecting inflammation or hepatic metabolism, pregnancy or lactation, recent acute infection, or incomplete clinical data. A total of 1,158 eligible patients were included, of whom 586 were diagnosed with MASLD and 572 were classified as non-MASLD according to guideline-defined diagnostic criteria.

This retrospective case–control study included adult patients who underwent clinical evaluation at a tertiary hospital in Tianjin, China, between November 2023 and October 2024. Patients were initially screened using International Classification of Diseases (ICD) codes, followed by manual chart review according to the predefined inclusion/exclusion criteria (**Figure 1**).

MASLD was diagnosed according to the 2024 Chinese Guideline for the Prevention and Treatment of Metabolic Dysfunction-Associated Fatty Liver Disease (31). Diagnostic criteria included: (1) Exclusion of excessive alcohol intake (≥210 g/week for men; ≥140 g/week for women); (2) Exclusion of secondary causes of steatosis (e.g., viral hepatitis, drug-induced liver injury, parenteral nutrition, Wilson’s disease, autoimmune liver disease); (3) Presence of at least one metabolic syndrome component (overweight/obesity, elevated blood pressure or hypertension, prediabetes or T2DM, hypertriglyceridemia, or reduced high-density lipoprotein cholesterol (HDL-C)); (4) The diagnostic criterion for hepatic steatosis was the presence of diffuse fatty liver or heterogeneous fatty liver on abdominal ultrasonography, or histological examination of liver biopsy showing macrovesicular or predominantly macrovesicular steatosis in ≥5% of hepatocytes, with or without non-specific hepatic inflammation.

Inclusion Criteria: (1) Age≥18 years (2) Diagnosis consistent with guideline-defined MASLD.

Exclusion Criteria: (1) Severe systemic disease (e.g., malignant tumors, advanced cardiovascular or renal dysfunction) (2) Use of medications affecting inflammation or hepatic metabolism (e.g., glucocorticoids, immunosuppressants, chemotherapy) (3) Pregnancy or lactation (4) Recent acute infection (5) Incomplete clinical data.

### 2.2 Data collection and laboratory measurements

Electronic medical records were retrospectively reviewed to obtain demographic and clinical data. Venous blood was drawn from all participants within 24 hours of hospital admission. A fully automated hematology analyzer (Sysmex XN-9000, Japan) was used to quantify neutrophils, lymphocytes, platelets, and red cell distribution width. Serum triglycerides(TG), total cholesterol (TC),HDL-C, low-density lipoprotein cholesterol (LDL-C), ALT, aspartate aminotransferase (AST) and γ-glutamyl transferase (GGT) were measured using a biochemical analyzer (Roche Cobas 8000, Germany). Inflammation–metabolic indices were derived as follows:

NHR: absolute neutrophil count / HDL-C concentration (32).

SIRI: monocyte count × neutrophil count / lymphocyte count (33).

### 2.3 Propensity score matching

To reduce baseline confounding, propensity score matching (PSM) (34) was performed using a logistic regression model with MASLD status as the dependent variable and age, sex, ethnicity, marital status, and comorbidities as covariates. A 1:1 nearest-neighbor matching algorithm without replacement was applied with a caliper of 0.02. Covariate balance was assessed using standardized mean differences (SMD), with SMD <0.1 indicating adequate balance.

### 2.4 Statistical analysis

All statistical analyses were performed using SPSS version 23.0 (IBM, Armonk, NY) and R (version 4.3.0). Normality was assessed using the Kolmogoro-Smirnov test. Continuous variables were summarized as mean ± SD or median (IQR) and compared using paired t-tests or Mann-Whitney U tests, as appropriate. Categorical variables were presented as frequencies (%) and compared using chi-square tests. After propensity score matching, conditional logistic regression models were used to evaluate the association of NHR, SIRI, and ALT with MASLD. Diagnostic performance was assessed using receiver operating characteristic (ROC) curves to calculate the area under the curve (AUC), optimal cutoff values, sensitivity, specificity, positive predictive value (PPV), and negative predictive value (NPV).

To further assess the calibration performance of the models, calibration curves, the Hosmer-Lemeshow test, and the Brier score were used. These metrics quantify the consistency between predicted probabilities and actual MASLD outcomes, objectively validating the model’s goodness-of-fit and discriminative reliability. Additionally, to compare the discriminative performance of different models, DeLong’s test was applied to evaluate the differences in the AUC between models. Net reclassification improvement (NRI) and integrated discrimination improvement (IDI) were used to quantify the classification gain brought by the combined models. These methods provide an objective assessment of the improvements in discriminative accuracy and clinical applicability.

### 2.5 Internal validation (bootstrap, 2,000 resamples)

To assess model stability and overfitting, internal validation was performed using 2,000 bootstrap resampling iterations following TRIPOD recommendations (35). In each iteration, a bootstrap sample was drawn with replacement, the model was refitted, and its performance was evaluated in both the bootstrap and original samples. Optimism was calculated as the difference in AUC between the bootstrap-fitted and test performance. The average optimism across 2,000 iterations was subtracted from the apparent AUC to obtain the optimism-corrected AUC. Calibration performance was assessed using the calibration intercept and slope, and a bootstrap-corrected calibration plot was generated. A two-sided *p*-value < 0.05 was considered statistically significant.

## 3 Results

### 3.1 Baseline characteristics after propensity score matching

**Figure 2:**
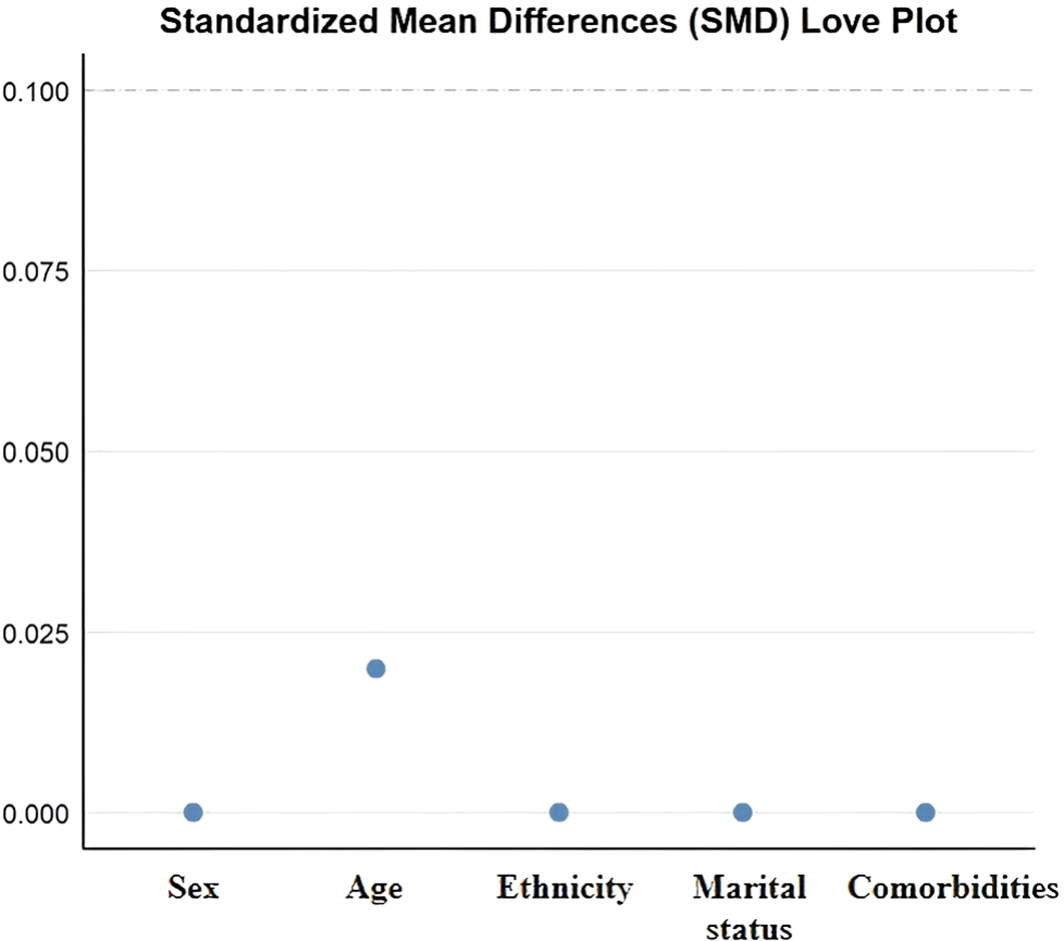
Love plot of standardized mean differences (SMDs) for covariates after propensity score matching. This plot displays the standardized mean differences of key baseline covariates including sex, age, ethnicity, marital status, and comorbidities—following propensity score matching. All covariates demonstrated SMD values close to zero, indicating excellent post-matching balance between the two groups.

A total of 586 individuals diagnosed with MASLD and 572 non-MASLD controls were initially screened. After propensity score matching, 522 participants were included, with 261 individuals in the MASLD group and 261 in the non-MASLD group. Baseline characteristics—including age distribution, sex, ethnicity, marital status, and comorbidities—were well balanced between the two groups, with no statistically significant differences (*P* > 0.05). SMDs for all covariates were <0.1, confirming adequate matching balance (**Table 1**, **Figure 2**).

**Table 1:** Comparison of general characteristics between the two groups before and after propensity score matching.

Variable	Before matching				After matching				
	MASLD (n=586)	Non-MASLD (n=572)	*χ* ^2^	*P*-value	MASLD (n=261)	Non-MASLD (n=261)	*χ* ^2^	*P*-value	SMD
Sex			5.377	0.020			0.000	1.000	0.000
Male	206(35.20%)	239(41.80%)			97(37.20%)	97(37.20%)			
Female	380(64.80%)	333(58.20%)			164(62.80%)	164(62.80%)			
Ethnicity			10.525	0.005			0.000	1.000	0.000
Han	580(99.00%)	552(96.50%)			258(98.90%)	258(98.90%)			
Hui	6(1.00%)	12 (2.10%)			3(1.10%)	3(1.10%)			
Manchu	——	8 (1.40%)			——	——			
Marital status			9.714	0.021			0.000	1.000	0.000
Married	486(82.90%)	476(83.20%)			221(84.70%)	221(84.70%)			
Unmarried	28(4.80%)	10(1.70%)			4(1.50%)	4(1.50%)			
Divorced	24(4.10%)	28(4.90%)			12(4.60%)	12(4.60%)			
Widowed	48(8.20%)	58(10.10%)			24(9.20%)	24(9.20%)			
Age			6.824	0.033			0.158	0.924	0.020
18–65 years	269(45.90%)	300(52.40%)			128(49.00%)	124(47.50%)			
66–79 years	282(48.10%)	251(43.90%)			125(47.90%)	128(49.00%)			
≥80 years	35(6.00%)	21(3.70%)			8(3.10%)	9(3.40%)			
Comorbidities			9.449	0.024			0.000	1.000	0.000
Coronary heart disease	318(54.30%)	360(62.90%)			164(62.80%)	164(62.80%)			
Diabetes	101(17.20%)	74(12.90%)			36(13.80%)	36(13.80%)			
Hypertension	56(9.60%)	44(7.70%)			41(15.70%)	41(15.70%)			
Stroke	111(18.90%)	94(16.40%)			20(7.70%)	20(7.70%)			

Categorical variables are presented as counts (percentages). *P* < 0.05 indicates statistical significance. A standardized mean difference (SMD) < 0.1 was considered to indicate good baseline balance after matching.

### 3.2 Comparison of inflammatory, lipid, and liver function parameters

Compared with the non-MASLD group, patients with MASLD exhibited significantly higher levels of TG, TC, LDL-C, ALT, AST, GGT, NHR, and SIRI (*P* < 0.05). In contrast, HDL-C levels were significantly lower in the MASLD group than in the non-MASLD group (*P* < 0.05 **Table 2**).

**Table 2:** Comparison of inflammation, lipid metabolism, and liver function parameters between the two groups.

Variable	MASLD (n=261)	Non-MASLD (n=261)	*Z*	*P*-value
TG (mmol/L)	1.73(1.26,2.48)	1.27(0.96,1.84)	-6.464	<0.001
TC (mmol/L)	4.430(3.77,5.22)	4.300(3.46,4.97)	-2.106	0.035
HDL-C (mmol/L)	0.970(0.85,1.11)	1.010(0.88,1.23)	-3.007	0.003
LDL-C (mmol/L)	2.460(1.91,2.99)	2.330(1.76,2.86)	-1.997	0.046
ALT(U/L)	19.00(14.00-29.00)	14.00(10.00-20.00)	-6.271	<0.001
AST(U/L)	17.00(14.00-22.00)	16.00(14.00-20.00)	-2.138	0.032
GGT(U/L)	25.00(17.50-37.50)	18.00(13.00-29.00)	-5.522	<0.001
NHR	6.28(4.65,8.83)	3.44(2.72,4.58)	-13.692	<0.001
SIRI	802.21(600.47,1150.71)	512.21(400.37,702.01)	-11.319	<0.001

Numerical variables are presented as median (lower quartile, upper quartile), Inter-group comparisons were performed using the Mann-Whitney U test, with the Z value as the test statistic. *P* < 0.05 indicates statistical significance. TG, triglycerides; TC,total cholesterol; HDL-C, high-density lipoprotein cholesterol; LDL-C, low-density lipoprotein cholesterol; ALT, alanine aminotransferase; AST, aspartate aminotransferase; GGT, gamma-glutamyl transferase; NHR, neutrophil-to-HDL-cholesterol ratio; SIRI, systemic inflammation response index; MASLD, metabolic dysfunction-associated steatotic liver disease.

### 3.3 Factors associated with MASLD

#### 3.3.1 Multicollinearity test

Initial collinearity analysis showed that the variance inflation factors (VIF) of TC (VIF = 10.676) and LDL-C (VIF = 8.116) were relatively high, indicating potential collinearity risks. To eliminate this interference, TC and LDL-C were removed, and collinearity tests were conducted again. The results showed that the VIF values for all remaining variables were less than 5, indicating no significant multicollinearity between the corrected variables (**Table 3**).

**Table 3:** Results of variance inflation factor testing.

Variable	Tolerance	VIF	Tolerance	VIF
TG	0.228	3.469	0.843	1.186
TC	0.094	10.676	——	——
HDL-C	0.528	1.895	0.758	1.319
LDL-C	0.123	8.116	——	——
ALT	0.651	1.537	0.653	1.532
AST	0.645	1.551	0.651	1.536
GGT	0.789	1.267	0.796	1.256
NHR	0.321	3.119	0.322	3.109
SIRI	0.362	2.759	0.364	2.744

The closer the tolerance value is to 1, the weaker the multicollinearity; a VIF > 10 indicates severe multicollinearity. In the initial tests, the VIF for total cholesterol (TC) and low-density lipoprotein cholesterol (LDL-C) were 10.676 and 8.116, respectively, indicating severe multicollinearity. Therefore, these two variables were excluded from subsequent analysis. After adjustment, the VIFs for the remaining variables were all < 5, indicating no significant multicollinearity, allowing them to be included in the subsequent multivariable model analysis. VIF, Variance Inflation Factor; TG, triglycerides; TC, Total Cholesterol; HDL-C, High-Density Lipoprotein Cholesterol; LDL-C, Low-Density Lipoprotein Cholesterol; ALT, alanine aminotransferase; AST, aspartate aminotransferase; GGT, gamma-glutamyl transferase; NHR, neutrophil-to-HDL-cholesterol ratio; SIRI, systemic inflammation response index; MASLD, Metabolic Dysfunction-Associated Steatotic Liver Disease.

#### 3.3.2 Univariate conditional logistic regression analysis

In univariable conditional logistic regression, TG, HDL-C, ALT,AST, GGT, NHR and SIRI were significantly associated with MASLD (*P* < 0.05; **Table 4**).

**Table 4:** Univariate logistic regression analysis of factors associated with MASLD.

Variable	Beta	*SE*	*Wald*	*OR (95%CI)*	*P*-value
TG (mmol/L)	0.180	0.080	5.113	1.198 (1.024–1.400)	0.024
HDL-C (mmol/L)	-1.5943	0.417	14.627	0.203 (0.090–0.460)	<0.001
ALT(U/L)	0.038	0.008	21.081	1.039 (1.022 –1.056)	<0.001
AST(U/L)	0.027	0.009	8.369	1.027 (1.008–1.046)	0.004
GGT(U/L)	0.009	0.003	6.325	1.009 (1.002–1.016)	0.012
NHR	0.731	0.096	58.405	2.076 (1.721–2.504)	<0.001
SIRI	2.654	0.354	56.158	14.216 (7.100 –28.462)	<0.001

Univariate conditional logistic regression analysis was used to assess the association between each indicator and MASLD. *P* < 0.05 indicates statistical significance. TG, triglycerides; HDL-C, High-Density Lipoprotein Cholesterol; ALT, alanine aminotransferase; AST, aspartate aminotransferase; GGT, gamma-glutamyl transferase; NHR, neutrophil-to-HDL-cholesterol ratio; SIRI, systemic inflammation response index; MASLD, Metabolic Dysfunction-Associated Steatotic Liver Disease.

#### 3.3.3 Multivariate conditional logistic regression analysis

**Figure 3:**
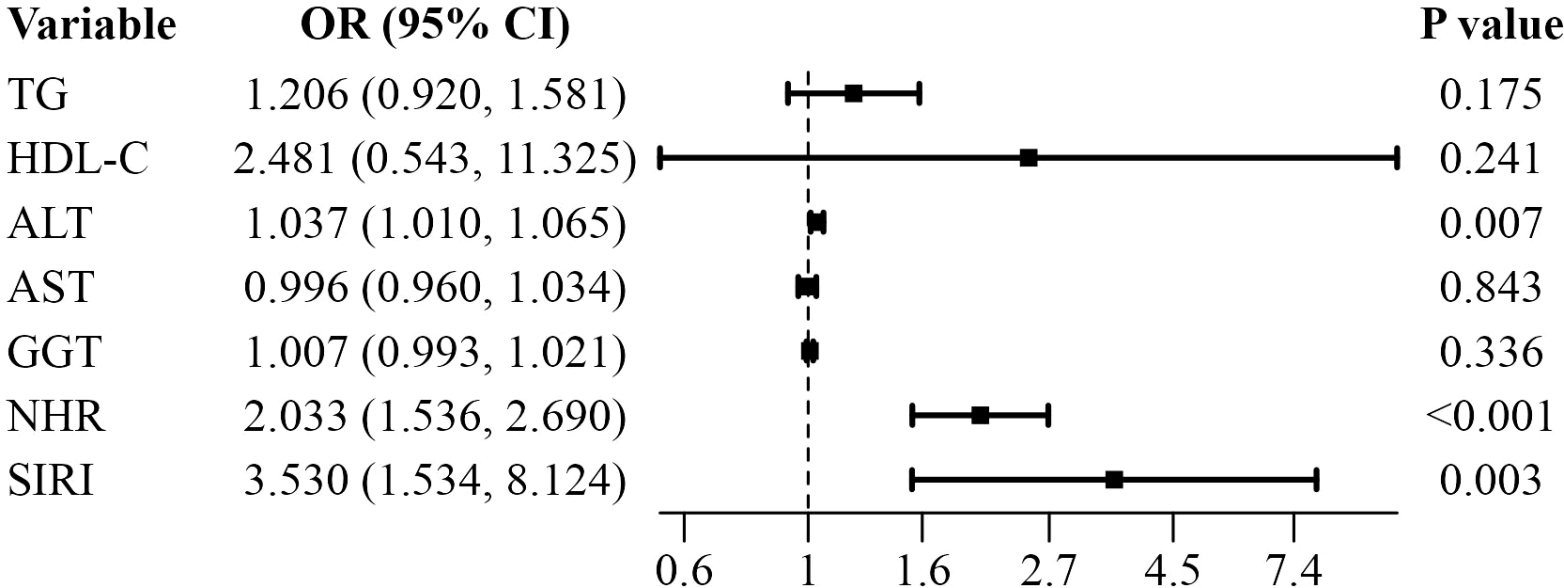
Multivariable associations between immune-related indices and MASLD. This forest plot displays the odds ratios (OR) and 95% confidence intervals (CI) for the association of each factor with MASLD, as determined by multivariable conditional logistic regression analysis. The variables included in the model are triglycerides (TG), high-density lipoprotein cholesterol (HDL-C), alanine aminotransferase (ALT), aspartate aminotransferase (AST), γ-glutamyl transferase (GGT), neutrophil-to-high-density lipoprotein cholesterol ratio (NHR), and systemic inflammation response index (SIRI). After adjusting for metabolic and liver-related variables, NHR and SIRI remained independently associated with MASLD.

In the multivariable model, ALT, NHR, and SIRI remained statistically significant. ALT: OR 1.037 (95% CI: 1.010–1.065), *P* = 0.007; NHR: OR 2.033 (95% CI: 1.536–2.260), *P* < 0.001; SIRI: OR 3.530 (95% CI: 1.534–8.124), *P* = 0.031 (**Figure 3**).

### 3.4 Evaluation of the performance of the combined model and internal validation

#### 3.4.1 Model discrimination and comparison of multiple models

**Figure 4:**
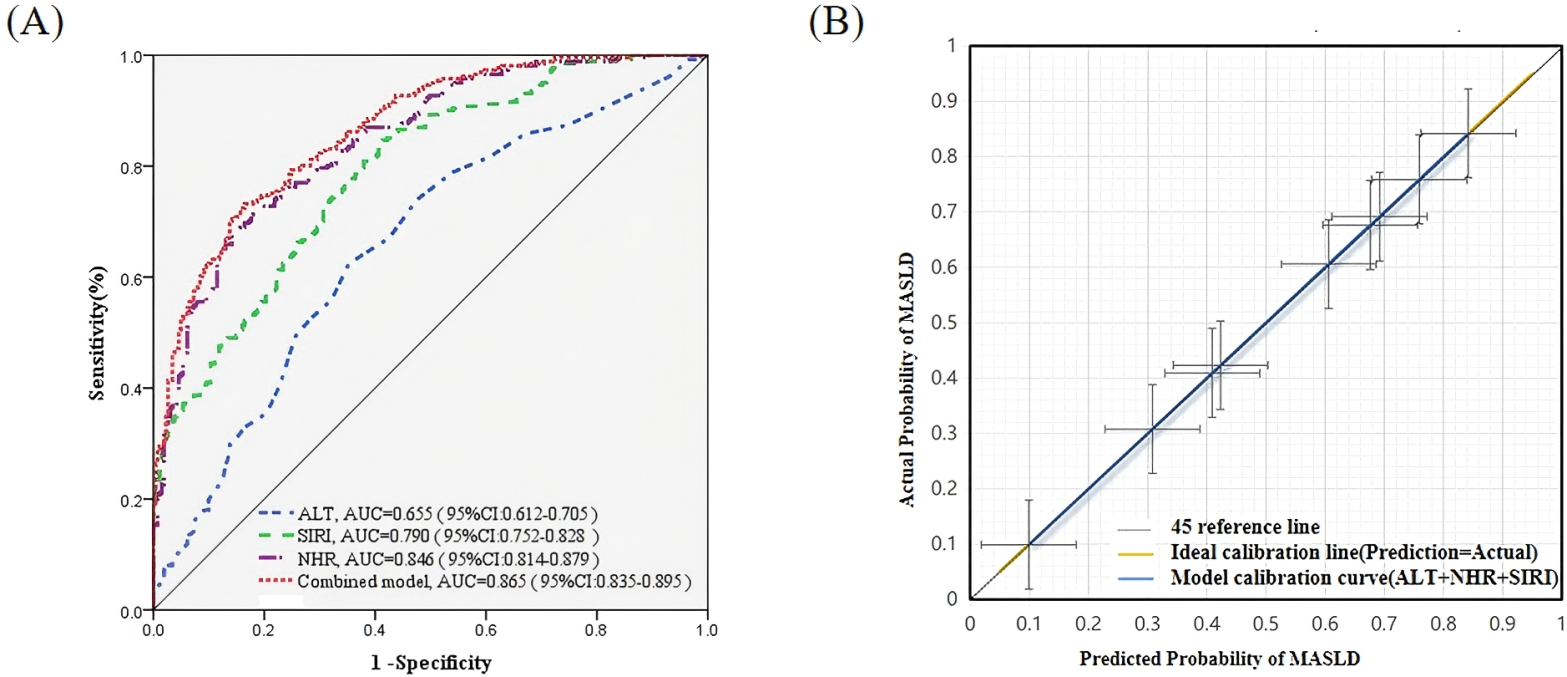
Diagnostic Performance and Calibration of the Combined Model for MASLD. **(A)** Receiver Operating Characteristic (ROC) Curves for ALT, SIRI, NHR, and the Combined Diagnostic Model in Predicting MASLD. The ROC curves show the diagnostic performance of alanine aminotransferase (ALT), the systemic inflammation response index (SIRI), the neutrophil-to-HDL cholesterol ratio (NHR), and the combined model (ALT + NHR + SIRI) in MASLD. The combined model achieved an area under the curve (AUC) of 0.865 (95% CI: 0.835–0.895), significantly outperforming the individual markers. **(B)** Calibration Curve for the Combined Model (ALT + NHR + SIRI). The yellow line represents the 45° ideal calibration line, while the blue line represents the model’s calibration curve. The error bars represent the 95% confidence interval for the actual incidence rate. This validates the alignment between the predicted probabilities and the actual incidence of MASLD. The model’s curve closely matches the 45° ideal reference line, indicating good calibration performance.

The results of the ROC analysis are shown in **Table 5** and **Figure 4**. Among the individual indicators, NHR demonstrated the best discriminative ability, with an AUC of 0.846, sensitivity of 68.20%, and specificity of 85.82%. SIRI also exhibited good diagnostic value, with an AUC of 0.790, sensitivity of 77.39%, and specificity of 65.51%. ALT showed moderate diagnostic accuracy, with an AUC of 0.655. The combined model consisting of NHR, SIRI, and ALT achieved the best diagnostic performance, with an AUC of 0.865, sensitivity of 72.41%, specificity of 84.30%, positive predictive value of 82.19%, and negative predictive value of 75.34%, indicating that the combined model outperforms individual markers in discriminative ability.

**Table 5:** Diagnostic performance of NHR, SIRI, ALT, and their combination for MASLD.

Variable	AUC	Youden Index	Cut-off Value	Sensitivity (%)	Specificity (%)	PPV (%)	NPV (%)	*P*-value
NHR	0.846	0.540	5.150	68.20	85.82	82.79	72.96	<0.001
SIRI	0.790	0.429	0.940	77.39	65.51	69.18	74.34	<0.001
ALT	0.655	0.268	16.000	62.45	64.36	63.67	63.15	<0.001
Combined model	0.865	0.567	——	72.41	84.30	82.19	75.34	<0.001

*P* < 0.05 indicates statistical significance.AUC, area under the receiver operating characteristic curve; PPV, positive predictive value; NPV, negative predictive value.ALT, alanine aminotransferase; NHR, neutrophil-to-HDL-cholesterol ratio; SIRI, systemic inflammation response index.

The DeLong test revealed that the difference in AUC between the single ALT model and the combined model was statistically significant (Z = -8.3083, *P* < 0.001). NRI analysis showed a total NRI of 0.345 (95% CI: 0.245–0.438), with a case group NRI^+^ of 0.264 (95% CI: 0.192–0.331) and a non-case group NRI^−^ of 0.081 (95% CI: 0.015–0.145). None of the 95% CI values crossed zero. IDI analysis showed a total IDI of 0.348 (95% CI: 0.282–0.424, *P* < 0.001), with the average predicted probability increasing by 0.174 in the MASLD group and decreasing by 0.174 in the non-MASLD group (*P* < 0.001) (**Table 6**). These results further confirm that the combined model significantly outperforms the single ALT model in both discriminative ability and risk stratification, demonstrating substantial incremental diagnostic value.

**Table 6:** Net reclassification improvement (NRI) and integrated discrimination improvement (IDI) of the ALT–NHR–SIRI combined model compared with the ALT-only model.

Metric	Point Estimate	SE	*95%CI*	*P*-value
Total NRI	0.345	0.049	0.245–0.438	——
NRI^+^	0.264	0.036	0.192– 0.331	——
NRI^−^	0.081	0.033	0.015–0.145	——
IDI	0.348	——	0.282–0.424	<0.001
ΔPred Prob (Cases)	0.174	——	0.139–0.216	<0.001
ΔPred Prob (Non-Cases)	-0.174	——	-0.214– -0.138	<0.001

P < 0.05 indicates statistical significance.

SE, standard error; CI, confidence interval; NRI, net reclassification improvement; IDI, integrated discrimination improvement; ΔPred Prob, mean change in predicted probability. Cases, patients with metabolic dysfunction-associated steatotic liver disease (MASLD); Non-Cases, non-MASLD control participants. All NRI and IDI values represent the incremental improvement in discriminative performance of the ALT–NHR–SIRI combined model relative to the ALT-only model. For NRI, statistical significance was determined by whether the 95% CI crossed 0; thus, P-values were not calculated. SE was not reported for IDI and ΔPred Prob.

#### 3.4.2 Evaluation of the original calibration of the model

The Hosmer-Lemeshow test result was χ² = 11.043, df = 8, *P* = 0.199 (*P* > 0.05), indicating that the predicted probabilities of the model closely fit the actual MASLD disease status. The Brier score was 0.151 (95% CI: 0.134–0.168), suggesting that the overall prediction error of the model was small. The calibration curve (**Figure 4**) visually demonstrates that the predicted probabilities closely align with the actual prevalence, with the curve closely following the 45° ideal line and the error band being narrow, further supporting the model’s good calibration performance.

#### 3.4.3 Internal validation results

**Figure 5:**
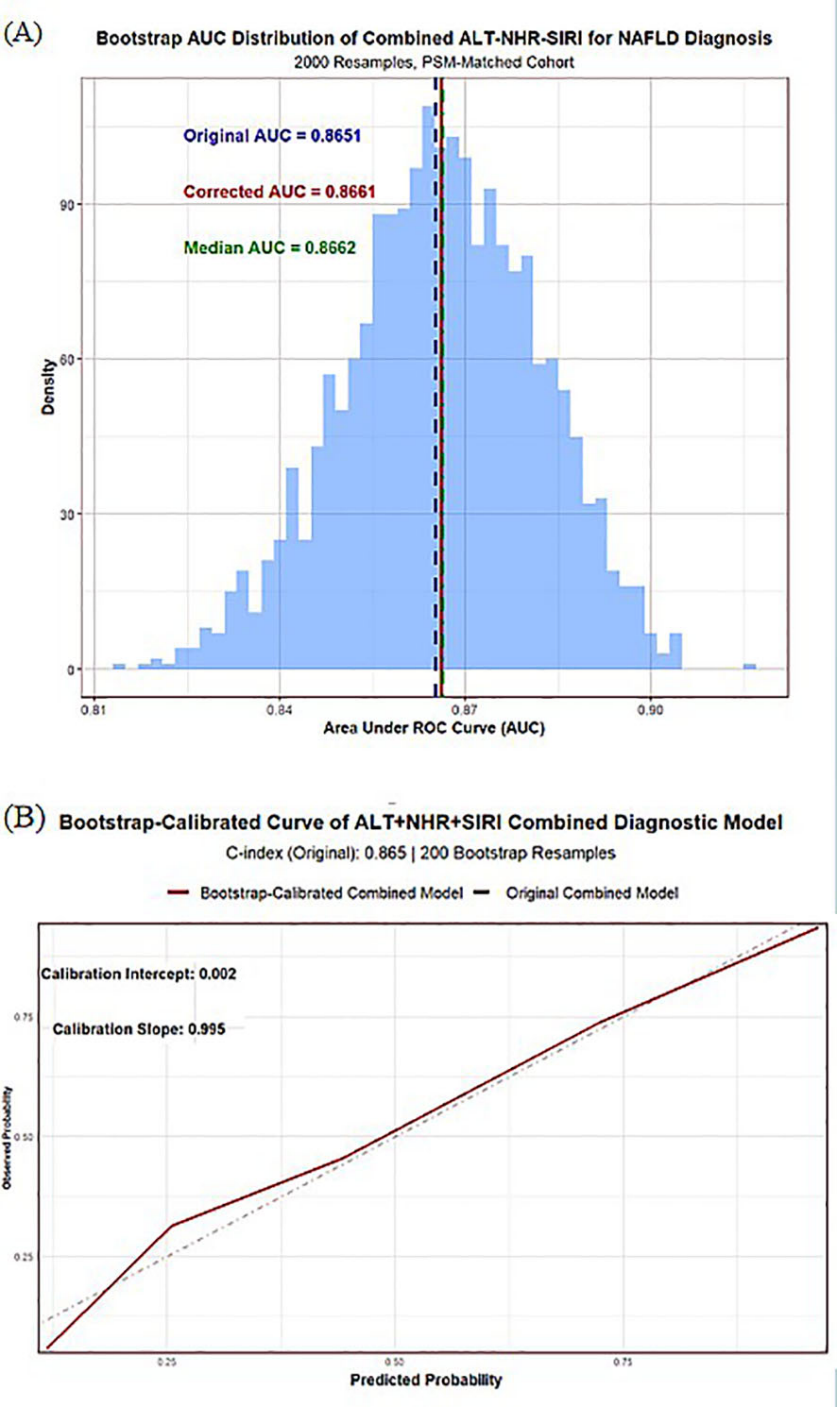
Internal validation of the combined ALT–NHR–SIRI diagnostic model using 2,000 bootstrap resamples. **(A)** Distribution of the bootstrap area under the ROC curve (AUC) estimates for the combined diagnostic model across 2,000 resamples. The original AUC was 0.8651, and the optimism-corrected AUC was 0.8662, with a median bootstrap AUC of 0.8662, indicating minimal overfitting and strong internal stability. **(B)** Bootstrap-calibrated curve demonstrating agreement between predicted and observed MASLD probabilities. The calibration intercept (0.002) and calibration slope (0.995) indicate excellent calibration and consistent model performance across risk levels.

Internal validation of the combined NHR-SIRI-ALT model is presented in **Figure 5**. The apparent area under the receiver operating characteristic curve (AUC) was 0.8651. After 2,000 bootstrap resamples, the optimism-corrected AUC was 0.8662, indicating virtually no reduction in model performance (**Figure 5A**). The mean optimism was −0.001, suggesting minimal overfitting. The bootstrap-corrected 95% confidence interval of the AUC ranged from 0.8363 to 0.8971.

As shown in **Figures 5B**, the bootstrap-corrected calibration curve demonstrated good agreement between predicted and observed MASLD probabilities across the full range of predicted risk, indicating satisfactory model calibration.

## 4 Discussion

In this propensity score matched study, we aimed to explore the independent associations of MASLD with two circulating immuno-metabolic inflammatory indices, NHR and SIRI. We also assessed the diagnostic discrimination performance of their combination with ALT for MASLD. After adjusting for key confounders such as age, sex, ethnicity, marital status, and comorbidities, and excluding multicollinearity interference from TC and LDL-C, both NHR and SIRI were independently associated with MASLD. The combined model incorporating all three indices demonstrated stable and excellent diagnostic discrimination ability. Internal validation using 2,000 bootstrap resamples showed no significant overfitting. These findings provide empirical evidence for the close association between systemic immune dysregulation and metabolic liver disease, and expand the application of immune-related non-invasive diagnostic markers in MASLD (10–13, 16).

### 4.1 Systemic immune activation as a core feature of MASLD

**Figure 6:**
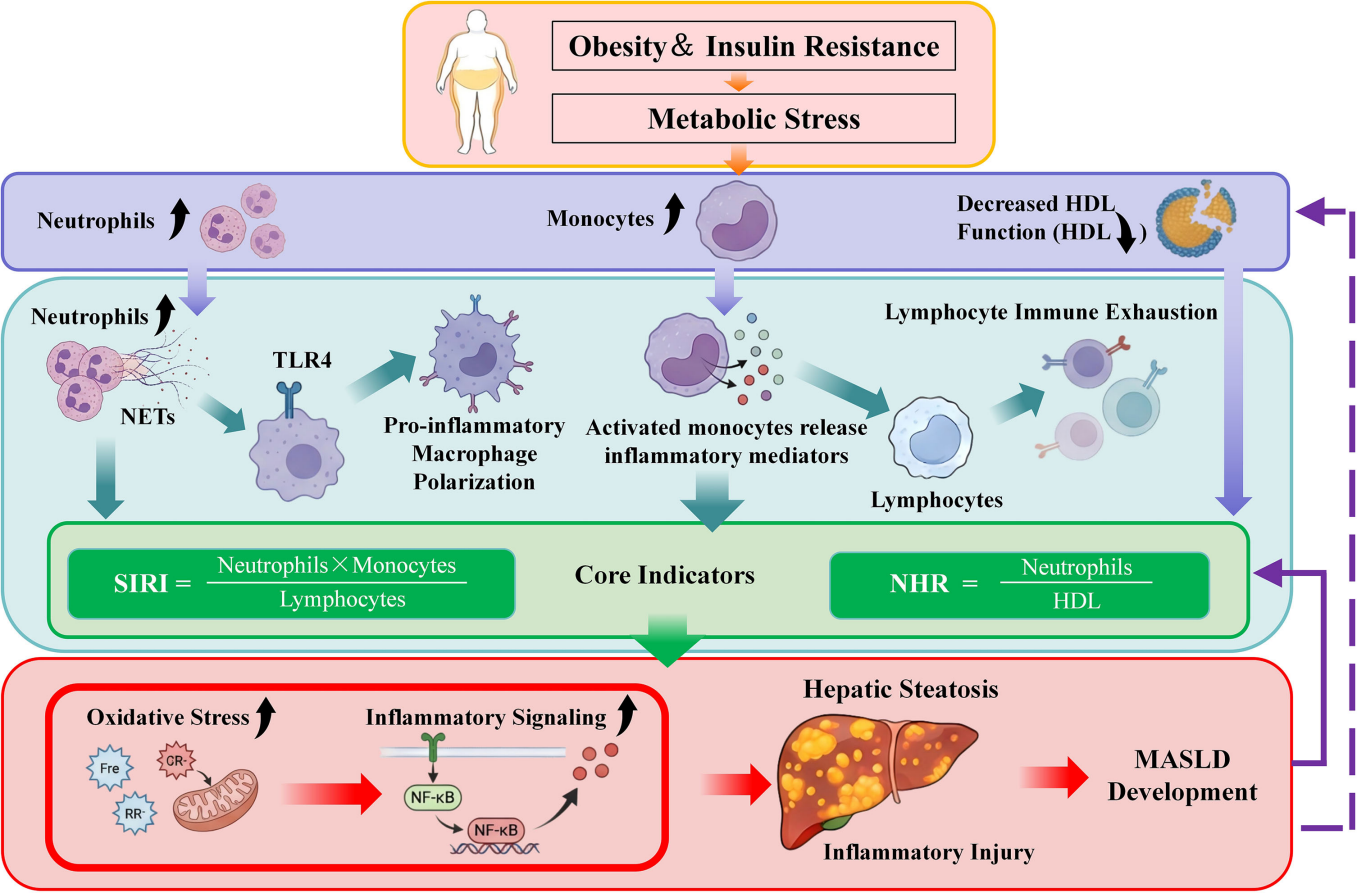
The immunometabolic mechanisms of NHR and SIRI in the development of MASLD. In Metabolic Dysfunction-Associated Steatotic Liver Disease (MASLD), the immunometabolic interaction framework between Neutrophil-to-High-Density Lipoprotein Cholesterol Ratio (NHR) and Systemic Inflammation Response Index (SIRI) is illustrated. Metabolic stress factors such as obesity and insulin resistance activate the immune system, particularly neutrophils and monocytes. These cells release inflammatory mediators, further promoting oxidative stress and the transmission of inflammatory signals, ultimately leading to hepatic steatosis and the progression of MASLD. NHR reflects the imbalance between neutrophil activation and HDL dysfunction, while SIRI reveals the imbalance between innate and adaptive immunity. The combined analysis of both provides deep insights into the immunometabolic mechanisms of MASLD and demonstrates their potential in early screening and non-invasive diagnosis of the disease. Neutrophil Extracellular Traps (NETs) Toll-Like Receptor 4 (TLR4) Nuclear Factor-kappa B (NF-κB) High-Density Lipoprotein (HDL) .

Our study provides data-driven support, rather than merely reiterating background concepts, further confirming the central role of systemic immune activation in MASLD. The independent association observed between SIRI and MASLD offers empirical support for the verifiable hypothesis that “imbalance between innate immune activation and adaptive immune regulation contributes to the development of MASLD”. The release of inflammatory mediators, reactive oxygen species, and neutrophil extracellular traps by activated neutrophils and monocytes amplifies inflammatory signals and exacerbates hepatocellular damage (36, 37). Additionally, alterations in lymphocyte subpopulations and immune exhaustion in MASLD patients lead to a persistent inflammatory response (38, 39). These immune changes collectively support the hypothesis that systemic immune dysregulation is associated with the development of MASLD (**Figure 6**), providing clinical evidence for further exploration of the disease’s immune-metabolic mechanisms.

### 4.2 SIRI as an indicator of systemic immune imbalance

SIRI integrates neutrophil, monocyte, and lymphocyte counts to reflect overall immune balance rather than isolated inflammatory signals (40). Previous studies have confirmed its prognostic value in inflammation-driven diseases, such as malignancies and cardiovascular diseases (41–43). This study extends its application to the field of metabolic liver disease.

In this study, SIRI demonstrated a sensitivity of 77.39% in identifying MASLD, suggesting that widespread immune activation may be detectable at the early stages of the disease. This finding aligns with experimental and clinical evidence, which indicates that immune dysregulation often precedes severe liver injury and may represent an early systemic response to metabolic stress (44). By simultaneously capturing both innate and adaptive immune components, SIRI provides unique information that cannot be conveyed by traditional inflammatory markers alone, further emphasizing the importance of coordinated immune regulation in the progression of metabolic liver diseases. However, this study did not directly validate the specific molecular mechanisms linking SIRI to MASLD. The above inferences are reasonable hypotheses based on existing evidence and still require further exploration through basic experimental research.

### 4.3 The neutrophil–HDL axis as an immuno-metabolic marker

The neutrophil-to-high-density lipoprotein cholesterol ratio (NHR) precisely captures the core imbalance between “enhanced pro-inflammatory activity of neutrophils” and “deficient anti-inflammatory function of HDL”. As a marker reflecting immune-metabolic stress, it has strong biological plausibility (45). In this study, NHR levels in MASLD patients were significantly higher than those in the control group, and it exhibited the strongest discriminative ability among all individual markers (with a specificity of 85.82%). This result leads to the following verifiable hypothesis: The functional imbalance of the neutrophil–HDL axis may be one of the core features of immune-metabolic dysregulation in MASLD patients, and the extent of this imbalance may reflect disease-related inflammatory burden.

This finding is consistent with several recent population studies, which have independently confirmed that NHR is positively correlated with both the risk of MASLD and the degree of liver fibrosis (46). However, it is important to clarify that the association analysis in this study only establishes a statistical correlation and cannot confirm the direct causal role of neutrophil–HDL axis imbalance in the pathogenesis of MASLD. Future targeted functional experiments are needed to further validate whether improving HDL’s anti-inflammatory function or inhibiting excessive neutrophil activation can alleviate hepatic steatosis and inflammatory damage in MASLD.

### 4.4 Complementary roles of NHR and SIRI: incremental discriminative value of the combined model

The synergistic effect of NHR and SIRI arises from their differential capture of immune-metabolic dysregulation. NHR focuses on the interaction between “innate immune activation and lipid-mediated immune regulation”, while SIRI emphasizes the balance between “innate immune activity and adaptive immune regulation”. Both indices are independently associated with MASLD, suggesting that multiple immune-metabolic pathways collectively contribute to the disease, providing a theoretical basis for their combined application.

The combined model integrating NHR, SIRI, and ALT significantly improved discriminative performance. It not only outperformed the ALT-only model but also surpassed the performance of individual markers, demonstrating clear incremental value. This improvement enhanced the accuracy of risk stratification while ensuring that predicted outcomes closely matched actual disease status. Internal validation confirmed excellent model stability with no significant overfitting, further supporting its reliability for clinical application.

The core significance of this complementary value lies in its ability to cover the multi-faceted pathological mechanisms of immune-metabolic dysregulation in MASLD. Metabolic stress driven by obesity and insulin resistance activates innate immunity and leads to HDL dysfunction, while simultaneously inducing adaptive immune exhaustion. By integrating these two key points, the combined model optimizes the disease’s discriminative ability and fills the gap in previous studies that lacked a systematic evaluation of their synergistic effects (46)(**Figure 6**).

### 4.5 Clinical implications

Both NHR and SIRI are derived from routine blood tests, offering advantages such as low cost and wide accessibility, making them suitable for primary care and metabolic disease specialty clinics. These indices are not intended to replace imaging or histological assessments but can help identify individuals who require further evaluation, in line with current MASLD management strategies (27).

Beyond their use in screening, NHR and SIRI reflect systemic immune activation, providing biological information about the disease beyond liver-specific markers. This further supports the concept that MASLD is a systemic immuno-metabolic disease, rather than a liver-limited condition (30). These markers hold potential for guiding the future development of therapeutic strategies targeting immune-lipid interactions.

### 4.6 Limitations and future directions

Several limitations need to be acknowledged. First, this is a single-center, retrospective case-control study of hospitalized patients, which may differ from the baseline characteristics of the community population, thus limiting the generalizability of the results. Second, although propensity score matching was used, residual confounding factors cannot be completely excluded. For instance, key variables such as BMI, diet, physical activity, and smoking status in electronic medical records were not fully documented, and some medication histories (e.g., statins, antidiabetic drugs, anti-inflammatory drugs) were not systematically recorded. Additionally, SIRI lacks specificity for MASLD, as its elevation may reflect systemic inflammation unrelated to metabolic liver disease (e.g., subclinical infection), and this factor should be considered when interpreting the results. Third, MASLD diagnosis was based on guideline-defined criteria and imaging studies, rather than histological assessments, limiting the ability to analyze the association between NHR/SIRI and disease severity (e.g., steatohepatitis or fibrosis). Fourth, the study lacked external validation, with model stability confirmed only through internal validation, limiting the generalizability of the model to other populations or institutions. Finally, as a correlation study, this research cannot establish a causal relationship between NHR/SIRI and MASLD; mechanistic inferences are limited to hypothesis generation.

Future research should adopt multi-center, prospective community cohort designs to validate the application value of NHR and SIRI in diverse populations. Longitudinal analyses are needed to clarify the association of these indices with the progression of MASLD and clinical outcomes. Further mechanistic studies should elucidate the interaction between immune activation and lipid dysfunction. External validation of the combined model in independent cohorts, along with subgroup analysis, should optimize its clinical application scenarios.

## 5 Conclusions

This study confirms that circulating immuno-metabolic inflammatory indices (NHR and SIRI) are independently associated with MASLD and each reflect distinct dimensions of hypothesized immune-metabolic dysregulation. When combined with ALT, they provide a stable, clinically accessible diagnostic method with significant incremental value for identifying the immune-metabolic risk of MASLD. In summary, these findings further strengthen the evidence for the central role of immune-inflammatory mechanisms in metabolic liver disease and highlight the potential value of immune-related biomarkers in clinical practice, particularly for early screening of high-risk populations in primary care settings.

## Data Availability

The raw data supporting the conclusions of this article will be made available by the authors, without undue reservation.
